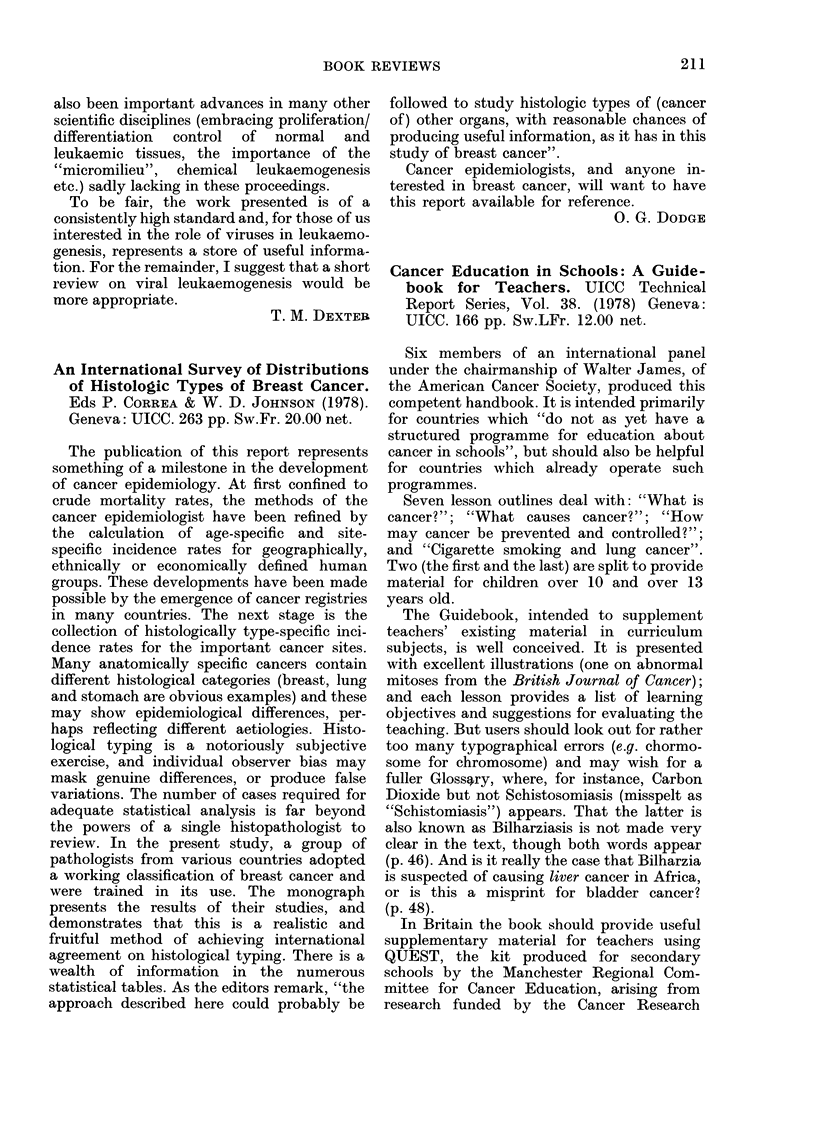# An International Survey of Distributions of Histologic Types of Breast Cancer

**Published:** 1979-02

**Authors:** O. G. Dodge


**An International Survey of Distributions of Histologic Types of Breast Cancer.** Eds P. Correa & W. D. Johnson (1978). Geneva: UICC. 263 pp. Sw.Fr. 20.00 net.

The publication of this report represents something of a milestone in the development of cancer epidemiology. At first confined to crude mortality rates, the methods of the cancer epidemiologist have been refined by the calculation of age-specific and site-specific incidence rates for geographically, ethnically or economically defined human groups. These developments have been made possible by the emergence of cancer registries in many countries. The next stage is the collection of histologically type-specific incidence rates for the important cancer sites. Many anatomically specific cancers contain different histological categories (breast, lung and stomach are obvious examples) and these may show epidemiological differences, perhaps reflecting different aetiologies. Histological typing is a notoriously subjective exercise, and individual observer bias may mask genuine differences, or produce false variations. The number of cases required for adequate statistical analysis is far beyond the powers of a single histopathologist to review. In the present study, a group of pathologists from various countries adopted a working classification of breast cancer and were trained in its use. The monograph presents the results of their studies, and demonstrates that this is a realistic and fruitful method of achieving international agreement on histological typing. There is a wealth of information in the numerous statistical tables. As the editors remark, "the approach described here could probably be followed to study histologic types of (cancer of) other organs, with reasonable chances of producing useful information, as it has in this study of breast cancer".

Cancer epidemiologists, and anyone interested in breast cancer, will want to have this report available for reference.

O. G. Dodge